# Endovascular repair of thoracic aortic disease with isolated left vertebral artery and unfavorable proximal landing zone using fenestrated castor stent-graft

**DOI:** 10.3389/fcvm.2023.1168180

**Published:** 2023-08-24

**Authors:** Zhenhua Wang, Changcun Fang, Han Song, Duoliang Wei, Xiangbin Meng, Xiao Bai, Chunxiao Liu, Xin Zhao

**Affiliations:** ^1^ Department of Cardiovascular Surgery, Qilu Hospital of Shandong University, Jinan, China; ^2^ Institute of Thoracoscopy in Cardiac Surgery, Shandong University

**Keywords:** isolated left vertebral artery, unfavorable proximal landing zone, castor single-branched stent-graft, fenestrated technique, thoracic aortic disease

## Abstract

**Objective:**

The main purpose of this study was to evaluate the safety and efficacy of Castor single-branched stent-graft combined with fenestrated technique in treatment of thoracic aortic disease (TAD) with unfavorable proximal landing area (PLZ) and isolated left vertebral artery (ILVA).

**Methods:**

From January 2018 to March 2022, 8 patients with TAD (6 patients with type B aortic dissections, 1 patient with type B intramural hematomas, and 1 patient with thoracic aortic aneurysm) underwent thoracic endovascular aortic repair with fenestrated Castor stent-graft due to the existence of ILVA and unfavorable PLZ. Demographic characteristics, surgical details, postoperative complications, follow-up and postoperative CTA imaging results were collected and analyzed.

**Results:**

The primary technical success rate was 100%. The mean operation time was 115 min (range, 70–180 min). All the left subclavian arteries (LSAs) and ILVAs of the eight patients were revascularized by fenestrated Castor stent-grafts. During the follow-up period, no deaths and complications were observed. No internal leakage, aortic rupture, retrograde type A dissection were found on computed tomography angiography. All of the LSAs and ILVAs maintained patency without stenosis.

**Conclusion:**

Castor single-branched stent-graft implantation combined with fenestration technique may be safe and feasible for TAD patients with ILVA and unfavorable PLZ.

## Introduction

Thoracic endovascular aortic repair (TEVAR) has been increasingly used for the treatment of various thoracic aortic disease (TAD), including type B aortic dissection (TBAD), intramural hematoma (IMH), penetrating ulcer, thoracic aortic aneurysm, traumatic aortic injury due to its advantages of high safety, less trauma and fewer postoperative complications (1). However, the success of TEVAR largely depends on the sealing of the proximal landing zone (PLZ), which requires that the length of the PLZ is at least 2.0 cm (2). Therefore, for patients with unfavorable PLZ, the proximal stent-graft inevitably needs to cover LSA. Isolated left vertebral artery (ILVA) directly originates from the aortic arch, usually between the left common carotid artery and left subclavian artery (LSA) (3). It is the second most common anatomical variant in the variation of the superior aortic trunk, with an incidence of 0.79%–8% (3–7). TAD with unfavorable PLZ and ILVA is rare. If the circle of Willis is incomplete and the LSA and ILVA are handled improperly, it may lead to an increased risk of postoperative complications such as subclavian steal syndrome, left upper limb ischemia and stroke (8,9).

The stented elephant trunk technique has been reported to treat complicated TBAD with ILVA and unfavorable PLZ (10), but high technical difficulty and mortality rate restrict its application. In addition, TEVAR combined with carotid-subclavian bypass (CSbp) and ILVA transposition was also widely used for treating this special condition, but the trauma was massive (11,12). Fortunately, methods for reconstructing supra-arch branches in TEVAR have developed rapidly, including chimney technique, periscope technique, fenestrated technique and single-branched stent-graft implantation. With these techniques, an adequate proximal landing area and supra-arch branches reconstruction could be realized. In this study, we shared our experience of using Castor single-branched stent-graft combined with fenestrated technique in treatment of TAD patients with ILVA and unfavorable PLZ, which may reduce the complexity and difficulty of surgery. We hope that our experience can provide an additional choice for cardiovascular surgeons to deal with this supra-arch variation, which may be less invasive, safe and effective.

## Materials and methods

**Figure 1:**
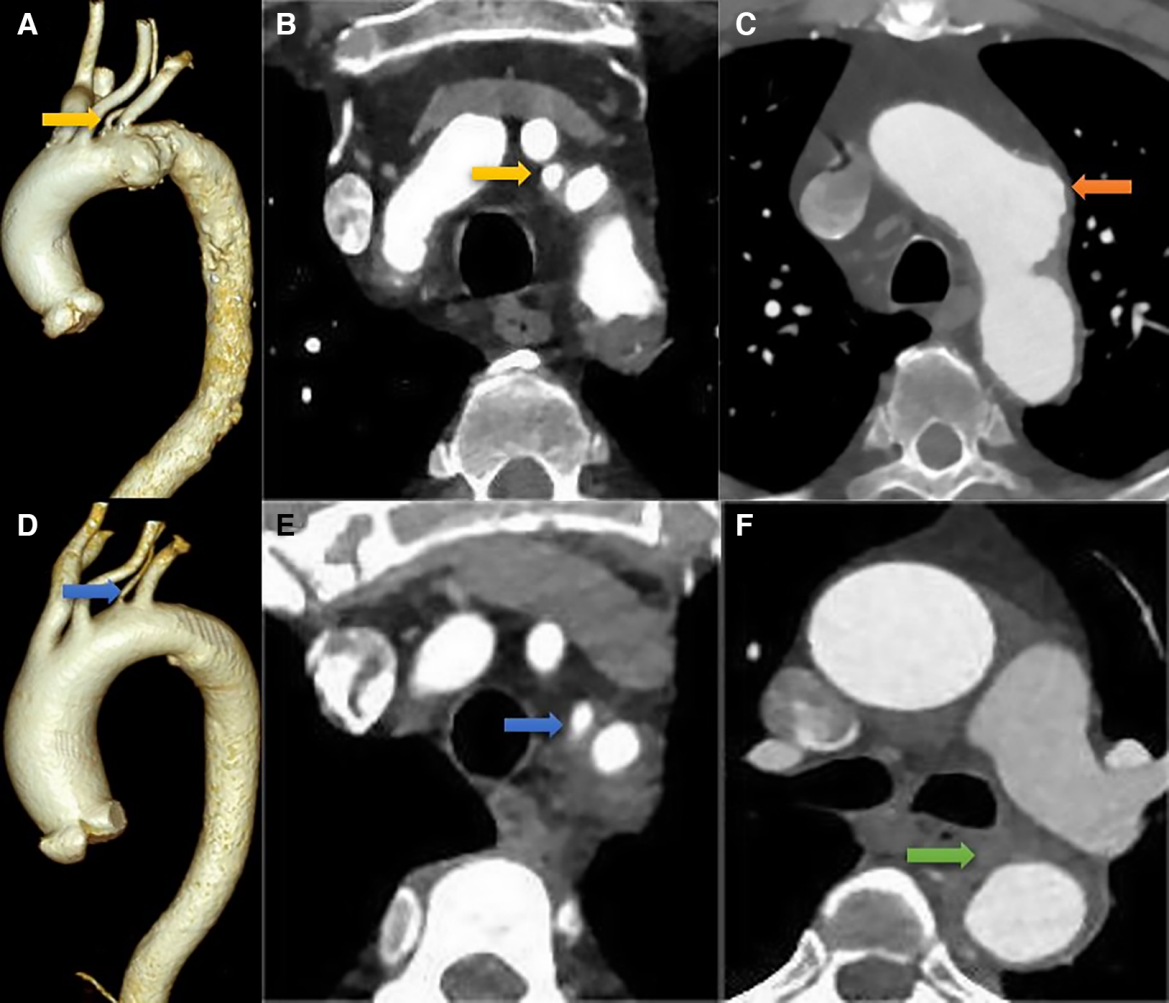
Preoperative CTA. (**A–C**) in a patient with thoracic aortic aneurysm, the ILVA (the yellow arrow) and aortic aneurysm (the orange arrow) are evident on the cross-sectional and three-dimensional reconstruction images; (**D–F**) in a patient with type B intramural hematoma, the ILVA (the blue arrow) and intramural hematoma (the green arrow) can be clearly seen from the cross-sectional and three-dimensional reconstruction images.

This is a single-center observational study. From January 2018 to March 2022, a total of 8 TAD patients with ILVA underwent TEVAR in our center (Figure 1). In this small series, there were 6 patients with TBAD, 1 patient with type B IMH, and 1 patient with thoracic aortic aneurysm. As the opening of LSA was involved by TAD in the eight patients, Castor single-branched stent-graft was applied to obtain adequate PLZ and reconstruct LSA. To reserve the ILVA, Castor single-branched stent-graft was fenestrated prior to implantation. Demographic characteristics, comorbidities, surgical details, postoperative conditions and follow-up results were collected and analyzed. This study was approved by the Ethics Committee of Shandong University Qilu Hospital. Because this is a retrospective study and the data are anonymous, we waived informed consent for this study.

### TEVAR procedure

**Figure 2:**
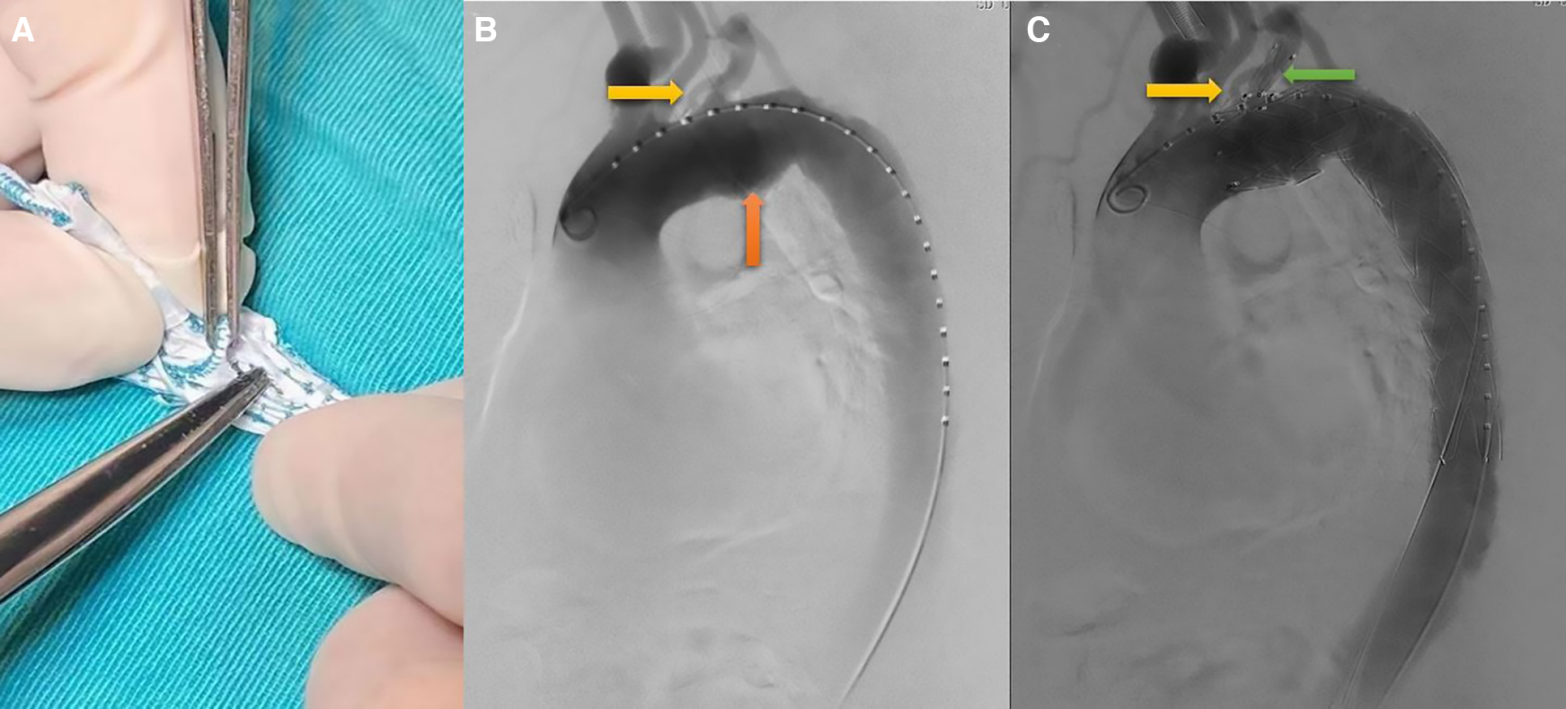
TEVAR procedure. (**A**) on-the-table fenestration; (**B**) preoperative angiography showed that isolated left vertebral artery (the yellow arrow) and aortic aneurysm (the orange arrow); (**C**) preoperative angiography showed that both ILVA (the yellow arrow) and LSA (the green arrow) were reconstructed.

The Castor stent-graft used in this study was the first single-branched stent-graft in China (9). It was with an integrated design of the main body and branch. All TEVAR procedures were performed under general anesthesia in the hybrid operating room by two experienced surgeons. The distance between LSA and ILVA, diameters of aortic zone 2, distal landing zone and the location of LSA and ILVA opening were obtained from preoperative computed tomography angiography (CTA). Usually, the right femoral artery (RFA) was chosen as the main approach. The details of the operation were as follows: first, the proximal part of Castor single-branched stent-graft was partially released from the sheath on the operation table. According to the aortic parameters obtained for preoperative CTA, including the relative location of LSA and ILVA, the size of ILVA opening, a fenestration was performed at an appropriate position (Figure 2A). The fenestration should be sutured firmly and circularly to achieve a smooth edge. The sutures around the stent-graft were tightened to avoid its deformation. The stent-graft was reinstalled and returned to the delivery sheath. Next, the RFA was exposed and two 6-F sheaths were inserted percutaneously into the left brachial artery (LBA) and left femoral artery (LFA), respectively. A 5-F pigtail catheter with a guidewire (catheter A) was pushed through the true lumen from the LFA to the ascending aorta. Angiography was subsequently performed to show the situation of the supra-arch branches and confirm the measurements obtained from CTA (Figure 2B). Then, another 5-F catheter (catheter B) was introduced from the LBA to the RFA through the true lumen along with a guidewire, and it was drawn out from the RFA and placed externally as a traction catheter. The third pigtail catheter with a guidewire (catheter C) was led from RFA to the ascending aorta and replaced with a super stiff guidewire. The traction wire of the branch was threaded into catheter B from RFA to LBA. Thereafter, the main body of the Castor single-branched stent-graft was delivered to the thoracic aorta along the super stiff guidewire, while the catheter B and the traction wire of the branch were moved simultaneously with the main body of delivery system. The position of the stent-graft was constantly adjusted to ensure the branch stent-graft was in good alignment with the LSA. Afterwards. the outer sheath and the soft sheath were removed, and the branch stent-graft was dragged into the LSA by pulling the traction wire. Finally, the main and branch grafts were released by pulling the trigger wire and the traction wire, respectively. Angiography was performed immediately to evaluate the stent-graft position, endoleak, LSA and ILVA perfusion (Figure 2C). All patients were given aspirin (100 mg/day) after operation to prevent thrombosis of the single-branched stent at least 6 months.

### Definition and main results

Primary technical success was defined as successful stent deployment without conversion to open surgery or death, patency of LSA with ILVA without significant stenosis, and no obvious signs of endoleak. Postoperative stroke was determined by brain CT or MRI scan. The main manifestations of spinal cord ischemia were distal weakness of lower limbs and intermittent claudication. The main symptoms of left upper limb ischemia were pulseless of left arm, intermittent claudication of left arm and cold shoulder sensation.

The follow-up results were obtained by telephone interview and at outpatient clinic visits. Primary outcomes during the follow-up included early (<30 days) survival, late survival, freedom from aortic-related mortality, the patency of LSA and ILVA. Furthermore, endoleak, retrograde type A dissection, aortic rupture, stent-graft fracture and displacement were also evaluated from postoperative CTA.

### Statistical analysis

Statistical analysis SPSS 26.0 (IBM, Armonk, New York, USA) was used for statistical analysis. Continuous variables are presented as mean ± SD and range. Categorical variables were presented as frequencies and percentages.

## Results

### Demographics and clinical characteristics

From January 2018 to March 2022, a total of 8 patients with TAD and ILVA underwent TEVAR in our center. Six (75.0%) patients were male, and the median age was 62.5 years old (range, 41 to 75 years old). The most common comorbidities are hypertension (62.5%). Four (50.0%) patients had smoking history, and 2 (25.0%) patients were diagnosed with COPD. According to preoperative CTA, there were 1 (12.5%) case of left vertebral artery dominant, 4 (50.0%) cases of right vertebral artery dominant, and 3 (37.5%) cases of symmetrical vertebral artery dominant. Additional details of patient characteristics were listed in Table 1.

**Table 1:** Baseline clinical characteristics of patients (*n*** **=** **8).

Variables	Median or No.	Range or %
Age, years	62.5	41–75
Gender, male	6	75
Hypertension	5	62.5
CAD	1	12.5
Diabetes	2	25.0
COPD	2	25.0
CKD	0	0
Smoking	4	50.0
Drinking	4	50.0
Type of arch		
Type I	3	37.5
Type II	4	50.0
Type III	1	12.5
Dominant vertebral artery		
Left dominant	1	12.5
Right dominant	4	50.0
Symmetrical dominant	3	37.5
Bovine aortic arch	0	0

CAD, coronary artery disease; CKD, chronic kidney disease; COPD, chronic obstructive pulmonary disease.

### Details of the procedure

TEVAR was performed in all patients, and the technical success rate was 100%. For each patient, the length of stent-graft main body was 200 mm and the length of the branch part was 25 mm. The median proximal stent-graft diameter was 34 mm (range, 30–36 mm). The median expansion ratio of proximal stents was 12.5% (range, 6.3%–15.4%). The median operation time was 115 min (range, 70–180 min). All the LASs and ILVAs were reconstructed. There was no endoleak during surgery.

### Early and late outcomes

The median duration of hospital stay was 8 days (range, 3–13 days). No postoperative complications were observed in the eight patients, including stoke, spinal cord ischemia, left upper limb ischemia, AKI and puncture complication. There were no in-hospital deaths.

**Figure 3:**
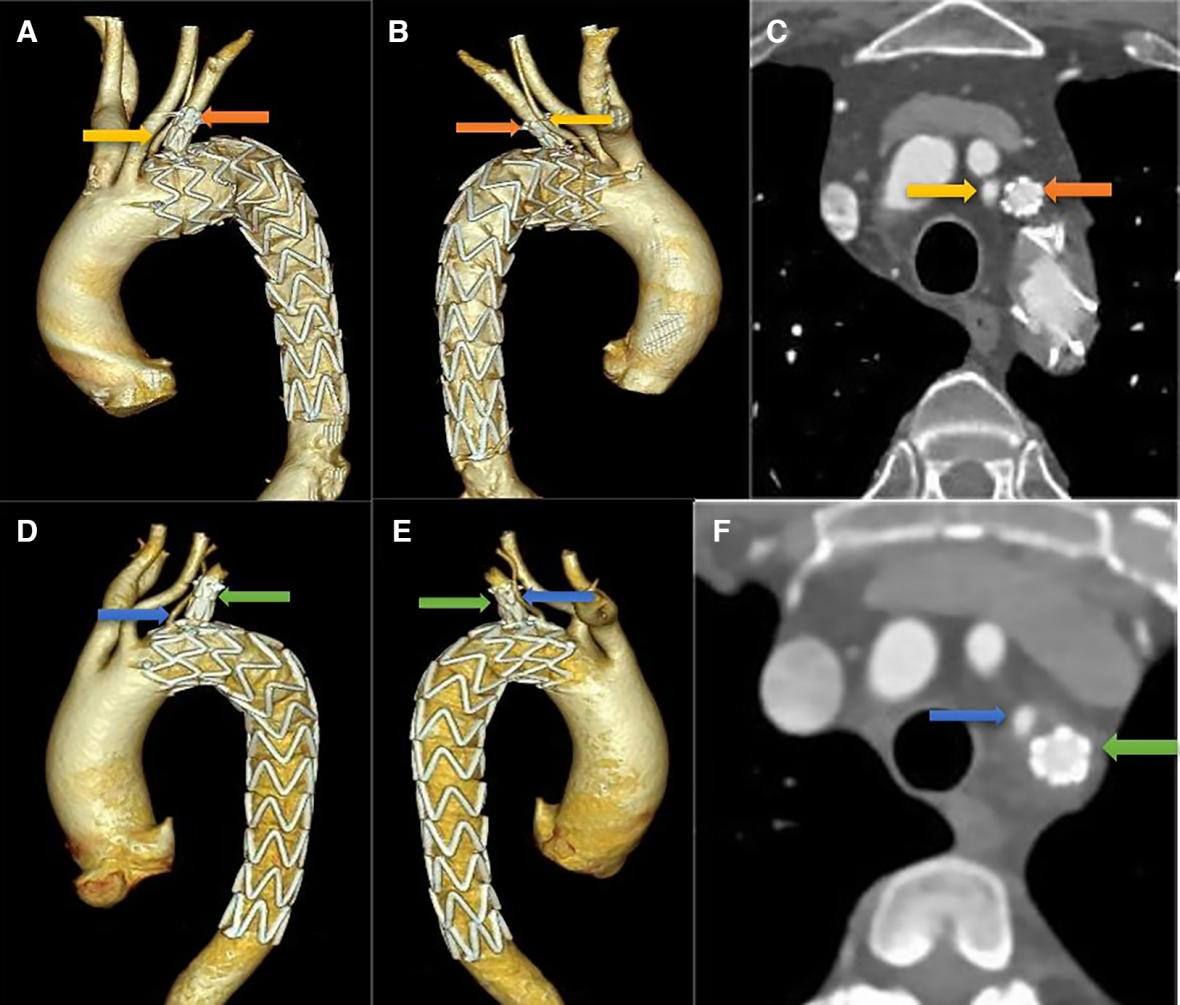
Follow-up CTA imaging. (**A–C**) in a patient with thoracic aortic aneurysm, both ILVA and LSA maintained patency without stenosis on three-dimensional reconstruction and cross-sectional images; (**D–F**) the same results can be seen in a patient with type B intramural hematoma.

The median duration of follow-up was 48 months (range, 6–72 months). No neurological complications were observed during follow-up. The long-term survival rate was 100%. All of the LSAs and ILVAs maintained patency without stenosis (Figure 3). In addition, no endoleak, aortic rupture, and retrograde type A aortic dissection were observed in the eight patients. None of them required further intervention. More details were shown in Table 2.

**Table 2:** Early and late outcomes of thoracic aortic disease with ILVA after TEVAR (*n* = 8).

Variables	Median or No.	Range or %
Early outcomes		
Technical success	8	100
Operation time, min	115	70–180
Proximal stent-graft diameter, mm	34	30–36
Expansion ratio of proximal stent	0.125	0.063–0.154
Immediate type IA endoleak	0	0
Hospital stays, days	8	3–13
Stroke	0	0
Spinal cord ischemia	0	0
Ischemic symptoms of the left arm	0	0
AKI	0	0
Puncture complication	0	0
Death	0	0
Late outcomes		
Follow-up, months	48	6–72
Paraplegia	0	0
Stroke	0	0
Reintervention	0	0
Death	0	0
Patency rate of LSA	8	100
Patency rate of ILVA	8	100
Retrograde type A dissection	0	0
Aortic rupture	0	0
Endoleak	0	0
Stent-graft fracture and displacement	0	0

AKI, acute kidney injury; ILVA, isolated left vertebral artery; LSA, left subclavian artery.

## Discussion

ILVA has been increasingly recognized as the second most common aortic arch branch variation, with an incidence of 0.79%–8% in general population (3–7). In patients with TBAD, the frequency of this anomaly is 3.6%, suggesting that ILVA may be related to the occurrence of TBAD (13). However, there were no clear guidelines whether ILVA should be reconstructed during surgery. In the most previous studies, the authors stated that the ILVA should be preserved (14,15). The left vertebral artery is an important component of the vertebrobasilar artery, which accounts for two fifths of the blood flow of the posterior cerebral artery (16). The posterior cerebral artery is the basic component of the Willis circle. It has been reported that the frequency of a complete Willis circle was 42% in the Western population. However, the frequency of a complete Willis circle in Chinese population was only 27% (17,18). Coverage of ILVA may increase the risk of spinal cord injury and postoperative stroke. However, some surgeons did not reconstruct the ILVA regularly (13). In their view, the ILVA was not necessarily reconstructed if it was with hypoplasia or thin size. Considering a high incidence of an incomplete circle of Willis in the Chinese population and the absence of cerebral artery CTA in an emergency, we regularly reconstructed the ILVA.

Several studies have reported the results of different surgical procedures for thoracic aortic disease and ILVA (10–12), including open surgery, hybrid surgery and total TEVAR. Zhu et al. (10) reported that seven patients with TBAD and ILVA underwent the stented elephant trunk procedure (open surgery). No deaths and postoperative complications were observed in the early term. Yang et al. (12) reviewed thirteen patients with thoracic aortic disease and ILVA using TEVAR with ILVA transposition and carotid-subclavian bypass (hybrid surgery). No deaths were observed in a mean follow-up of 22 months (range, 13–29 months). No complications were observed during the follow-up, including neurologic deficits, bypass occlusion and ILVA stenosis. Open surgery and hybrid surgery had shown satisfactory outcomes in treating the patients with thoracic aortic disease and ILVA. However, considering the massive trauma of these surgical methods, we attempted a less invasive approach, which is total TEVAR.

Total TEVAR for the thoracic aortic disease has been the mainstream treatment for its encouraging outcomes and less invasiveness. A length of 2.0 cm PLZ is essential for the success of total TEVAR. However, total TEVAR of thoracic aortic disease with ILVA and unfavorable PLZ was rarely reported. Ding et al. (13) reported 9 TBAD patients with ILVA treated with zone-2 TEVAR combined with LSA chimney technique. In order not to cover ILVA, the chimney stents were all bare stents. Neurologic deficits and chimney stent occlusions were not observed during surgery and follow-up, but complications such as immediate type I endoleak, type II endoleak and occlusion of the ILVA origin occurred. In our study, Castor single-branched stent-graft implantation combined with fenestration technique was used for treating patients with thoracic disease and ILVA. During the follow-up, all of the LSAs and ILVAs maintained patency without stenosis, no endoleak, aortic rupture and retrograde type A aortic dissection were observed in the eight patients. Based on excellent results, Castor single-branched stent-graft combined with fenestrated technique may be safe and feasible for repairing thoracic aortic disease with ILVA and insufficient PLZ.

It is considered that fenestrated or single-branched stent technique may lead to the risk of endoleak due to groove formation (12). However, previous published studies showed that the incidence of endoleak after Castor single-branched stent-graft implantation was low (19,20). Actually, single-branched stent-graft is more consistent with the design concept of anatomic correction. The anchoring effect of its branch part reduces the risk of stent displacement and endoleak (21). In 2004, McWilliams et al. first applied the in-situ fenestration technique of a thoracic stent-graft. to preserve LSA and demonstrated its feasibility (22). Since then, fenestration techniques have been developed continuously, mainly including in-situ fenestration and on-the-table fenestration. Currently, this technique has been applied in clinical practice through several preoperative and intraoperative methods classified as (1) mechanical, such as wires and hollow needles, and (2) physical, including laser and radiofrequency perforation (23). The incidence of endoleak in the early stage of fenestration is 0%–3.2% described in the previous study (24). In fact, in cases of aortic arch disease that involved only the inner curve of the aorta, the selection of on-the-table fenestration technique can help reduce the incidence of endoleak after TEVAR (25). However, in our study, no endoleak of fenestration was observed during surgery and follow-up, which may be due to the small size of fenestration and healthy aortic wall surrounding the ILVA opening. Although the fenestrated technique may affect the long-term durability of the stent-graft, the early and mid-term results are still satisfactory whether in external pre-fenestration or in-situ fenestration (24–26). It is worth mentioning that computational fluid dynamics method based on patient-specific CTA images has been widely applied to evaluate the efficacy of complex TEVAR (27). Quantitative analysis of computational hemodynamics would be conducive to revealing the physiological effect of Castor single-branched stent-graft on the thoracic aortic disease with unfavorable PLZ and ILVA. Therefore, we are considering a related computational hemodynamics study in eight patients in the future.

Of course, our study still has some limitations. First, it is a single-center retrospective study with a small sample size. Second, the follow-up time was relatively short, and the long-term effect still needs further follow-up. Third, our study lacks relevant study on computational fluid dynamics, which needs to be further improved in the future. Finally, we lacked a comparison with open or hybrid surgery. These features and the limited experience with these anatomical variants do not allow us to make definitive recommendations from clinical or technical aspects. However, the surgical approach we have employed has hardly been reported in previous studies. We hope that larger registries or studies will confirm its feasibility and safety in addressing this challenge in the future.

## Conclusion

Our limited experience indicates that single-branch stent-graft combined with fenestrated technique may be a safe and feasible treatment for TAD with ILVA and unfavorable PLZ. It can provide surgeons with an attractive choice due to its less invasiveness and good short-term effects. However, the durability and long-term effects of the technique needs to be further evaluated. Future studies with larger sample sizes and longer follow-up are warranted to confirm this finding.

## Data Availability

The original contributions presented in the study are included in the article/Supplementary Material, further inquiries can be directed to the corresponding author.
